# Flexibility of PCNA-Protein Interface Accommodates Differential Binding Partners

**DOI:** 10.1371/journal.pone.0102481

**Published:** 2014-07-18

**Authors:** Anthony M. Pedley, Markus A. Lill, V. Jo Davisson

**Affiliations:** Department of Medicinal Chemistry and Molecular Pharmacology, Purdue University, West Lafayette, Indiana, United States of America; Wake Forest University, United States of America

## Abstract

The expanding roles of PCNA in functional assembly of DNA replication and repair complexes motivated investigation of the structural and dynamic properties guiding specificity of PCNA-protein interactions. A series of biochemical and computational analyses were combined to evaluate the PIP Box recognition features impacting complex formation. The results indicate subtle differences in topological and molecular descriptors distinguishing both affinity and stoichiometry of binding among PCNA-peptide complexes through cooperative effects. These features were validated using peptide mimics of p85α and Akt, two previously unreported PCNA binding partners. This study characterizes for the first time a reverse PIP Box interaction with PCNA. Small molecule ligand binding at the PIP Box interaction site confirmed the adaptive nature of the protein in dictating overall shape and implicates allosterism in transmitting biological effects.

## Introduction

PCNA has emerged as an essential protein to promote formation of numerous protein complexes in order to regulate cellular processes associated with a DNA damage response [1]–[3]. Regulation and formation of specific PCNA-protein complexes are highly coordinated processes involving combinations of post-translational modifications and accessory proteins aimed at preserving genomic stability [4]. The expanded roles for PCNA are being revealed from an increasing list of functionally diverse interacting nuclear proteins [1], [5], [6]. The traditional view of PCNA as a simple processivity factor is being replaced by increasing knowledge of the context-dependency of PCNA complexes that implicate changes in structural features to accommodate functional properties. While the structural scaffold concept holds true for understanding how these proteins interact with PCNA, regulation of the complexes depends on the conformation of the binding partner(s) and potentially on the conformation of PCNA itself.

Initial studies investigating how proteins interact with PCNA have identified a variety of conserved sequence motifs and topological relationships that select among these interactions. Mutagenesis and structural data identified a PCNA-protein interaction site proximal to the interdomain connecting loop (IDCL) that accommodates the anchoring of the PIP Box, a conserved sequence binding motif [5]. The PIP Box motif is characterized by QXXφXXΩΩ, where φ is any hydrophobic residue, Ω is any aromatic residue, and X is any amino acid. Initial studies that established the importance of the PIP Box interaction with PCNA used a C-terminal peptide modeled after the amino acid sequence of p21. Peptides lacking the PIP Box sequence motif or specific residues exhibited significant decreases in overall binding [7]–[9]. High-resolution structural analyses by X-ray crystallography also indicate similarities in the overall binding mode of several PIP Boxes [10]–[13].

Protein complexes are routinely formed for regulation of a number of cellular processes to maintain cellular homeostasis. The formation of these complexes requires both molecular recognition through conformation selection or induced fit [14]. Molecular recognition accounts for the initial selection and binding of a ligand to form a transition state of protein-ligand complex. Recent studies have indicated that selection of binding partners can be dictated by the pre-existing conformation of the ligand [15]–[17]. After recognition, a higher affinity complex is generated through optimization of side chains and backbone conformations that enhance the overall complex stability. The study of these processes has shown to be useful in understanding selectivity in protein complex formation and has led to the design and optimization of novel small molecule inhibitors [18]–[20].

Despite structural similarities among the PIP Box sequences, p21 and FEN1 PIP Box peptides showed almost a 1000-fold difference in binding affinity, as determined by isothermal titration calorimetry (ITC) [7], [9], [10], [21]. Further, the p21 PIP Box peptide was able to compete with FEN1 for binding and inhibit *in vitro* SV40 DNA replication [7], [9]. NMR spectroscopy studies of PCNA-protein complexes indicate that restrained flexibility within the N-terminus, IDCL, and C-terminus may be attributed to different affinities and molecular interactions amongst binding partners [22]. Together, these results suggest that other molecular features may contribute to the overall binding between PCNA and those binding partners containing a PIP Box.

This study pursues understanding of the molecular recognition elements that participate in forming specific PCNA-protein complexes at the PIP Box interaction site. In the process, the local structural features and the extent to which these elements aid in the stabilization of complexes are evaluated in the context of small molecule binding interactions. To establish minimal features for binding, a series of five peptide mimics of known PIP Box containing PCNA binding partners demonstrated significant differences in binding. Molecular dynamics simulations of these complexes were used to interrogate the PIP Box interaction site to understand the subtle structural alterations that may be necessary and/or sufficient for overall affinity. These descriptors were queried amongst other peptides that contain a similar conserved sequence motif to identify potentially new PCNA interacting proteins. Last, the ability to translate these recognition elements to small molecule design and optimization was performed after studying the disruption of PCNA-POGO ligase (PL) interactions with a recently reported small molecule antagonist. These data further probe the extent to which PCNA is capable of selective interactions with binding partners through distinct conformations and offer insights for modulating these complexes.

## Materials and Methods

All supplies and reagents were purchased through Sigma Aldrich and used without further purification unless otherwise noted. All computational calculations were performed on a Red Hat Linux platform with an Intel quad-core processor. All computational models were rendered in PyMOL [23].

### Ligation Independent Cloning of PCNA Construct

Ligation independent cloning compatible expression vector pEV-L8 containing an N-terminal (His)_6_-tag and TEV protease recognition site was linearized by digestion with Ssp1 (New England BioLabs), purified by gel filtration, and treated with T4 DNA polymerase (Novagen) in the presence of dGTP (New England BioLabs) for 30 min at 22°C followed by heat inactivation at 75°C for 20 min. The *PCNA* fragment was amplified by PCR from a template plasmid (Genecoepia) using a high fidelity polymerase Platinum *Pfx* DNA polymerase (Invitrogen). The resulting PCNA products were treated with T4 DNA polymerase in the presence of dCTP (New England BioLabs) to generate 5′ overhangs necessary for annealing. A total of 0.2 pmol of each insert was incubated with 0.01 pmol of pEV-L8 vector in 3 µL reaction mix at 22°C for 10 min followed by addition of 1 µL of 25 mM EDTA for 22°C for 5 min. Annealing reaction products were transformed into X10Gold competent cells (Strategene) and plated on LB agar containing 50 µg/mL kanamycin. Individual colonies were grown and the constructs were assessed by PCR for insert size and verified by sequencing before propagating the plasmid.

### Expression and Induction of Recombinant PCNA Protein

A 10 µL aliquot of chemically competent BL21(DE3) E.coli cells (Agilent) were transformed by heat shock with 1 µL of purified plasmid encoding the fusion protein N-terminal (His)_6_-PCNA for 30 sec at 42°C. Cells were immediately placed on ice for 2 minutes and 140 µL of SOC was added. Transformed cells were allowed to grow for 1 h at 37°C before streaking on a LB agar plate containing 50 µg/mL kanamycin. Single isolated colonies were picked and a added to a 1 L culture of LB broth containing 50 µg/mL kanamycin. The culture was grown at 37°C until an OD in 1.0 before inducing with 0.4 mM IPTG for 4 h at 37°C. Cultures were centrifuged at 4000×*g* for 20 min at 4°C.

### Purification of Recombinant PCNA Protein

Transformed cells were resuspended in 40 mL of iced cold lysis buffer (50 mM Tris HCl at pH 8.0, 0.15 M NaCl) on ice. Cells were lysed on ice by sonication at a 30% amp output for 3 min (20 sec pulses) and lysate was centrifuged at 4000×*g* for 20 min at 4°C. Lysate was purified by affinity chromatography. Soluble protein lysate was added to Ni-NTA resin (ThermoScientific) and incubated for 2 h at 4°C. The resin was centrifuged at 800×*g* for 5 min at 4°C and the resin was washed 10×1.5 mL with wash buffer (50 mM Tris HCl at pH 8.0, 0.15 mM NaCl, 20 mM imidazole). Protein was eluted off of the resin with 50 mM Tris HCl at pH 8.0, 0.15 mM NaCl, 1 M imidazole. Eluted protein was monitored by Bradford dye (BioRad), pooled, and exposed to final concentrations of 1 mM DTT, 1 mM EDTA, and 0.5 M ammonium sulfate. Protein was incubated for 1 h at 4°C before extensive dialysis against 25 mM HEPES at pH 7.4, 10% glycerol, 0.01% Triton X-100. Dialyzed protein was centrifuged at 2000×*g* to remove any precipitated protein. Purity was determined by SDS-PAGE and monomer concentration was determined by UV-Vis at 280 nm using a molar extinction coefficient of 14,800 M^−1^cm^−1^.

### Solid Phase Peptide Synthesis

Solid phase peptide synthesis was performed on CLEAR Amide resin (Peptides International, 100 µmol, 0.30 mmol equiv/g resin). Fmoc-protected amino acids (Anaspec) and resin were deprotected using 20% piperidine in DMF for 20 min. Fmoc-protected amino acids (5 equiv) were coupled under standard peptide synthesis conditions [O-benzotriazole-N,N,N′,N′-tetramethyluronium hexafluorophosphate (5 equiv), diisopropylethylamine (10 equiv)] for 1 h at room temperature. Resin was washed 6 times with DMF after each coupling and deprotection step. Reactions were monitored through a ninhydrin (Kaiser's) test. For FITC labeling of peptides, the immobilized peptide was subjected to coupling and deprotection of Fmoc-N-6-aminohexanoic acid (Anaspec) prior to labeling with a 1.1 molar excess of fluorescein isothiocyanate isomer I dissolved in 500 µL of a 12∶7∶5 pyridine/dichloromethane/DMF solution and added to the resin for 16 h in the dark. After completion of the peptide, the resin was washed 6 times with DMF and 2 times with dichloromethane and dried on vacuum for 15 min. Peptides were cleaved off the resin using 95∶2.5∶2.5 trifluoroacetic acid/triisopropylsilane/water solution for 4 h at room temperature and precipitated into iced cold diethyl ether. Peptides were purified by HPLC using a gradient elution of acetonitrile in water containing 0.1% trifluoroacetic acid over 30 min. Peptide molecular weight and sequence were validated on an Applied Biosystems MALDI-TOF/TOF 4800 mass analyzer.

### Fluorescence Polarization Binding Assay

Increasing amounts of recombinant PCNA prepared in binding buffer (25 mM HEPES at pH 7.4, 10% glycerol, 0.01% Triton X-100) were added to 50 nM FITC-PL peptide and incubated at room temperature for 30 min. Binding assays were analyzed by fluorescence polarization on a DTX880 Multimode plate reader (Beckman Coulter) using an excitation of 485 nm and an emission filter at 535 nm at 30°C. Anisotropy values (*N* = 4) were statistically evaluated using Grubbs' test for outliers at a significance level of 0.05. Anisotropy values were represented as mean ± standard error of mean (*Y*) and plotted as a function of the logarithm of monomeric PCNA protein concentration (*X*). Data was fit to a non-linear regression model in Origin 8.6 using Eq. (1) to calculate a dissociation constant (*K_d_*).
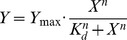
(1)where *n* is the Hill slope.

For Z′ factor analysis, solutions of 50 nM FITC-PL peptide in the presence and absence of 1 µM recombinant PCNA protein (monomer concentration) were incubated for 30 min at room temperature (*N* = 24) under the same detection conditions. Raw anisotropy values were statistically evaluated using Grubbs' test for outliers at a significance level of 0.05 and represented as the mean ± standard deviation (*Y*) of each condition (*X*). The Z′ factor was calculated using Eq. (2).
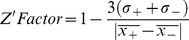
(2)where *σ_+_* and *σ_−_* refer to the standard deviations of the positive (FITC-PL peptide in the presence of PCNA) and negative (FITC-PL peptide only) controls, 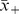 and 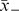 represent the mean of the positive and negative controls [24].

### Fluorescence Polarization Competition Assay

Increasing amounts of 12 amino acid competitive peptide ligand were originally prepared in water and diluted into binding buffer (25 mM HEPES at pH 7.4, 10% glycerol, 0.01% Triton X-100) and incubated with 50 nM N-terminal FITC-labeled PL peptide and 1 µM recombinant PCNA protein (monomeric concentration) for 30 minutes at room temperature. Competition assays were analyzed by fluorescence polarization on a DTX880 Multimode plate reader (Beckman Coulter) using an excitation of 485 nm and an emission filter at 535 nm at 30°C. Anisotropy values (*N* = 4) were statistically evaluated using Grubbs' test for outliers at a significance level of 0.05 and converted to fraction of FITC-PL peptide bound (*f_b_*) using Eq. (3) and represented as mean ± standard error of mean (*Y*). Fraction of the FITC-PL peptide bound (*Y*) was plotted as a function of the logarithm of the 12 amino acid peptide antagonist concentration (*X*), and *IC_50_* values were determined by fitting the data to Eq. (4) in Origin 8.6.
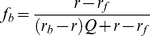
(3)where *r* represents the observed anisotropy value, *r_f_* represents the anisotropy value of the FITC-PL peptide only, *r_b_* represents the anisotropy value of the FITC-PL peptide in the presence of saturated PCNA protein, and *Q* is the ratio of quantum yield of the bound (*q_b_*) and free (*q_f_*) FITC-PL peptide (*Q = q_b_/q_f_*).
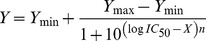
(4)where *n* is the Hill slope.

Dissociation constants for the competitive ligands (*K_i_*) were calculated using a modified form of the Cheng-Prusoff equation, Eq. (5), previously reported for fluorescence polarization assays [25].
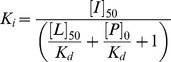
(5)where *[I]_50_* is the concentration of the 12 amino acid competitive peptide at 50% inhibition, *[L]_50_* is the concentration of the FITC-PL peptide at 50% inhibition, *[P]_0_* is the monomeric concentration of the PCNA protein at 0% inhibition, and *K_d_* is the dissociation constant calculated from Eq. (1).

Anisotropy values were also analyzed using complete and incomplete binding models without non-specific effects as outlined in Roehrl *et al.* [26]. Briefly for complete binding, the fraction of the FITC-PL peptide bound (*Y*), as determined by Eq. (3) was plotted as a function of total competitive ligand concentration, *L_T_*, (*X*) and fit in Origin 8.6 to Eq. (6) to determine *K_d2_*, an estimated dissociation constant of the competitive ligand-PCNA interactions. 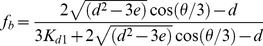
(6)where
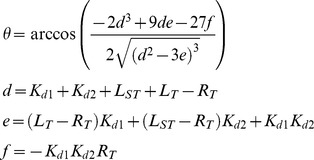



For incomplete binding (when *f_b_*≠0 at [peptide]_max_), the fraction of the FITC-PL peptide bound (*Y*), as determined by Eq. (3) was plotted as a function of total competitive ligand concentration, *L_T_*, (*X*) and fit to the implicit Eq. (7) in Origin 9.1 to determine *K_d2_* and *K_d3_*, estimated dissociation constants for a four state model described in Roehrl *et al.* [26]. 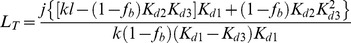
(7)where
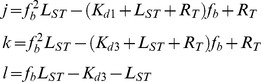
and *K_d1_* is the dissociation constant of the PCNA-PL interaction determined by Eq. (1), *L_ST_* is the concentration of the FITC-PL peptide ligand, *R_T_* is the monomeric concentration of PCNA.

### Molecular Dynamics Simulations

PCNA-peptide and apo-PCNA models were constructed from the PCNA-PL co-crystal structure (PDB 1VYJ). PL was removed from the co-crystal structure to yield an apo-PCNA model. Molecular threading of the PL peptide was performed to reflect desired peptide sequences. PCNA-peptide complexes were minimized in Desmond [27] with the OPLS 2005 force field and solvated with TI3P water model in the presence of 0.15 M sodium chloride buffer. An orthorhombic water box was generated with a 10 Å buffer region, all overlapping water molecules were removed, and the system was neutralized in the presence of sodium cations.

All molecular dynamics simulations were carried out in Desmond using the OPLS 2005 force field and TI3P solvent model in the presence of 0.15 M sodium chloride buffer [27]. Long-range electrostatic interactions were determined using a smooth particle mesh Ewald method with a grid spacing of 0.8 Å. For non-bonded van der Waals interactions, a cut off of 9.0 Å was set. All simulations were performed for 5.0 ns using the Desmond NPT method with a six step slow relaxation protocol prior to the molecular dynamics run: (i) 2000 step limited-memory Broyden-Fletcher-Goldfarb-Shanno (L-BFGS) minimization with a loose convergence restraint of 50 kcal/mol/Å; (ii) 2000 step L-BFGS minimization with a convergence constraint of 5 kcal/mol/Å; (iii) a 12 ps Berendsen NVT simulation at a temperature of 10 K with restraints on solute heavy atoms; (iv) a 12 ps Berendsen NPT ensemble at a temperature of 10 K and pressure at 1.01325 bar with restraints on solute heavy atoms; (v) a 24 ps Berendsen NPT ensemble at a temperature of 300 K and a pressure at 1.01325 bar with restraints on solute heavy atoms; (vi) a 24 ps Berendsen NPT ensemble at a temperature of 300 K and a pressure at 1.01325 bar with restraints on residues beyond 15 Å of the restrained ligand. The 5.0 ns molecular dynamic simulation run was performed using NPT ensemble. Temperature of the simulation was kept at 300 K using a Nosé-Hoover thermostat. Pressure was maintained at 1.01325 bar using the Martyna-Tobias-Klein method. Energy and trajectory data was recorded at every 1.2 ps and 5.0 ps, respectively.

Trajectory data were processed after removal of the peptide ligand. The resulting Cα atoms were aligned to the first frame of the simulation using the analysis tools in Desmond to generate RMSD and Cα fluctuations (RMSF) values and visualized in VMD [28]. For principal component analysis, PCNA residues 258–261 were removed to avoid inclusion of extreme terminal motions. The Cα atoms (*N* = 257) were aligned to the first frame of the simulation in VMD. Principal component analysis was performed using the Bio3d R package [29] to analyze conformational differences between the aligned trajectory snapshots between 4.5–5.0 ns (100 per PCNA-ligand simulation). The first two orthogonal eigenvectors (principal components) generated were plotted on the same set of axes. Average trajectory coordinates at RMSD convergence (4.5–5.0 ns) was performed using AMBER12 [30]. Anchor residues and corresponding estimates of free energy of binding was performed using ANCHOR, http://structure.pitt.edu/anchor/ [31].

## Results

### Characterization of PL-PCNA Interactions

**Figure 1:**
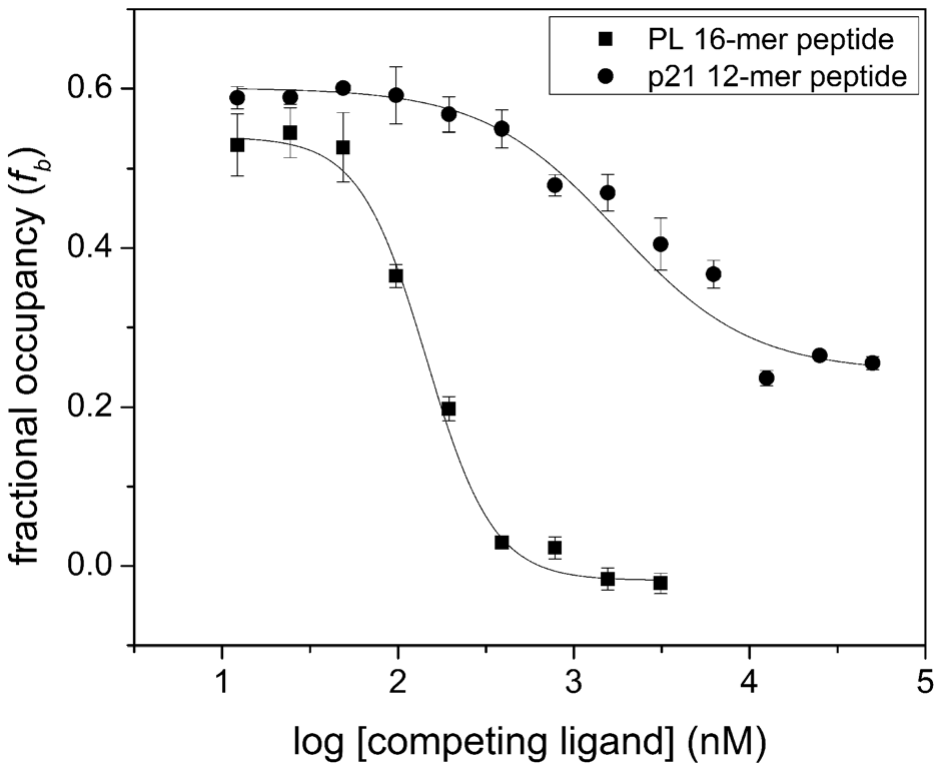
PIP Box Peptide Competition of PCNA-PL Interactions by Fluorescence Polarization. Competition of 50-PL and 1 µM recombinant PCNA protein (monomer concentration) with increasing amounts of unlabeled PL (squares) and p21 (circles) peptides. Anisotropy values (*N* = 4) were converted to fractional occupancy, *f_b_*, values using Eq. (3) and represented as mean ± standard error of mean (SEM). Error bars associated with specific data points may be within the data points themselves.

Prior studies involving the design and evaluation of peptide ligands using the PIP Box recognition sequence have been based upon a high affinity 20-mer sequence from the tumor suppressor p21 [7]. More recently, POGO-ligase (PL), a hybrid 16-mer peptide modeled after the PIP Box conserved sequence of DNA ligase and POGO DNA transposase, demonstrated a similar binding affinity to PCNA by ITC [7]. In addition to the available X-ray co-crystal structure with PCNA, this peptide was shown to inhibit PCNA-dependent DNA replication *in vitro* [7], [13]. To further characterize PL binding to PCNA, a fluorescence polarization assay was pursued [32]. An N-terminal fluorescein isothiocyanate (FITC) labeled PL peptide was synthesized and incubated with increasing concentrations of PCNA to afford a *K_d_* = 760±19 nM (Fig. S1). Based on these data, the anisotropy of free FITC-PL peptide, *r_f_*, and FITC-PL peptide in the presence of saturating monomeric concentrations of PCNA, *r_b_*, were 0.0470±0.0011 and 0.1587±0.0029, respectively. For self-competition studies, increasing concentrations of the unlabeled PL peptide was incubated with 50 nM FITC-PL peptide and 1 µM PCNA (monomer concentration) as shown in Figure 1. Under those conditions, 56.8% of the FITC-PL peptide was bound to PCNA. The unlabeled PL peptide yielded an *IC_50_* = 144±13 nM with an estimated *K_i_* = 136 nM as determined by the Cheng-Prusoff equation [33], which is similar to the *K_d_* experimentally determined for PL binding to PCNA by ITC [7]. The lower affinity observed with the fluorescent version of the PL peptide suggests that despite the addition of an aliphatic linker at the N-terminus of the peptide, the fluorophore does influence the peptide interaction with PCNA.

### Competition of Short PIP Box Peptide Mimics to Disrupt PL-PCNA Interactions

The fluorescence polarization competition assay was implemented for comparing a series of novel, shorter (12-mer) peptide sequences that share a minimal PIP Box motif and interact within a defined sub-structure region of human PCNA. Using the molecular interaction sites of PCNA observed in the co-crystal p21 peptide X-ray structure (PDB 1AXC) and the putative functional interactions of PIP Box containing proteins, four different 12-amino acid peptides were synthesized (Table 1). These peptide mimics were modeled after DNA polymerase δ, p21, Abl, and Mcl-1. A peptide modeled after the p66 subunit of DNA polymerase δ and a C-terminal p21 peptide have previously been shown to interact at PCNA-PIP Box, and it was anticipated that they should exhibit some degree of competition [10], [12]. A peptide modeled after Abl was chosen because it has been reported to bind PCNA and contain a classic PIP Box conserved sequence motif [34]. The fourth peptide was modeled after Mcl-1, an apoptotic regulatory protein that has been reported to contain a PIP Box. Recent studies have suggested that it does not participate in direct binding to PCNA without the assembly of additional accessory proteins [22]. For these studies, the previously identified Mcl-1 PIP Box-like peptide was selected as a negative control.

**Table 1:** PIP Box Containing Peptide Mimic Experimental and Simulation Data.

Ligand	PIP Box Peptide Sequence^a^	RMSD (Å)	*IC_50_* (µM)	*K_i_* (µM)^b^	Hill Slope	*f_b_* at [peptide]_max_
(apo)		2.377	---	---	---	---
PL	SAVL**Q**KK**I**TD**YF**HPKK	1.666	0.145±0.013	0.136	−2.3±0.3	0.00
p21	RR**Q**TS**M**TD**FY**HS	1.800	1.8±0.5	0.477	−1.1±0.3	0.25
DNA polymerase δ (p66 subunit)	NR**Q**VS**I**TG**FF**QR	2.279	67.9±15.8	28.9	−1.2±0.2	0.00
Abl	PG**Q**RS**I**SLR**Y**EG	2.238	71.3±12.1	30.4	−2.6±0.7	0.10
Mcl-1	GV**Q**RNHETA**F**QG	---	*nd*	*nd*	*nd*	*nd*
PI3K (p85α subunit)	TL**Q**YL**L**KH**FF**KL	2.556	7.5±0.3	2.9	−3.4±0.5	0.00
Akt	HR**FF**AG**I**VW**Q**HV	1.690	8.5±4.2	3.4	−1.3±0.3	0.06
T3	---	---	19.0±4.2	7.8	−1.6±0.6	0.11

a amino acid residues shown in bold represent PIP Box conserved sequence motif residues.

b determined based on Eq. (3) in Nikolovska-Coleska *et al.* [25].

*nd* =  no competition detected.

Prior to competition studies, the reproducibility of the anisotropy values of 50 nM FITC-PL peptide in the presence and absence of 1 µM PCNA (monomer concentration) was assessed. A Z′ factor of 0.650 was calculated (Fig. S2). The ratio of quantum yields for the bound and free states of the FITC-PL peptide was 0.70 indicating that upon binding, the fluorescence of the FITC-PL peptide is quenched. For competition studies, increasing concentrations of the 12-mer competitive peptide ligand was incubated with 50 nM FITC-PL peptide and 1 µM PCNA (monomer concentration) and fraction of the FITC-PL peptide bound, *f_b_*, to PCNA was calculated directly from anisotropy values. The p21 peptide mimic was shown to bind with a *K_i_* of 477 nM derived from the fitted *IC_50_* values by a modified form of the Cheng-Prusoff equation [25]. The observed inhibitory constant for the p21 12-mer peptide was similar to the *K_d_* = 640 nM reported by ITC for a similar (non-identical) p21 12-mer PIP Box containing peptide at the same assay temperature [7], [22]. Competition with the p66 and Abl 12-mer peptides resulted in inhibitory constants of 28.9 µM and 30.4 µM, respectively. Consistent with NMR studies, the Mcl-1 peptide mimic did not bind recombinant PCNA in this competition assay (Table 1) [22].

### Stoichiometry of PCNA-Peptide Complexes

PCNA in solution exists as a dynamic equilibrium, where the trimer is considered the functional state of the protein [3], [35], [36]. The approximate *K_d_* of the PCNA trimer was determined to be 21 nM, and the trimeric state is observed predominantly at concentrations above 100 nM [37]. Given the concentration of PCNA protein used in the fluorescence polarization assay was 48-fold greater than the approximate dissociation constant, there is reason to expect that three binding sites exist within the PCNA trimer in solution and that partial displacement and cooperative effects are possible (Table 1). The fraction bound of the PL was directly revealed from the anisotropy values and Hill slopes were calculated based upon fits of the primary binding data to Eq. (4). In the case of the self-competition with the control PL peptide, complete displacement was observed consistent with full occupancy. (Fig. 1, S3). However, the best fits for competitive models indicate variation in the binding mechanisms. In the case of the higher affinity p21 peptide, the displacement of the PL labeled peptide was partial at high competitive ligand concentrations (Fig. 1, S3). An alternative method for data analysis was employed to evaluate the differences in the peptide binding with PCNA. A more general approach that can distinguish basic models was implemented for comparisons [26]. The implicit fitting to Eq. (7) was consistent with an incomplete binding model for the p21 peptide; this was not the case for the PL, p66 or Abl peptides (Table S1, Fig. S4). The observation of mechanistic ambiguity may illustrate an important point about PCNA complex formation. These results suggest that binding of a single site on the PCNA trimer in solution results in distinct conformations depending upon the peptide sequence. For the case of p21-PCNA complex, there is a conformation that favors binding of the PL peptide likely in the same trimer.

### Characterization of New Peptide-based PIP Box Interaction Probes

To test the models, previously unreported PIP Box containing peptides were screened from the existing human genome (Table 2). The screen focused on the primary sequence of proteins to assess whether additional PIP Box-like sequences are capable of binding PCNA. PIP Box peptide mimics (12-mers) of two proteins, PI3-kinase (p85α regulatory domain) and Akt, were generated for further analysis. These two proteins were shown to exist as a complex in the nucleus of U2OS cells and facilitate the phosphorylation and dissociation of p21 from PCNA and loading onto DNA [39]. However, Akt was observed to have an atypical reverse-PIP Box, which has only been reported to exist in one other PCNA binding partner, 3-methyladenine DNA glycosylase [40]. To date, the significance of any reverse PIP Box interaction has not been previously characterized. The Akt and p85α peptides effectively competed with PL for binding to PCNA showing *K_i_* of 3.4 µM and 2.9 µM, respectively (Table 1). In both cases, the displacement of the labeled-PL peptide appeared complete. While consistent with the model, a robust fit to the general model for complete displacement was difficult in both cases (Table S1, Fig. S4) [26]. The Hill slope for binding of the p85α peptide to PCNA was determined to be −3.4±0.5 which is distinct from the reverse-PIP Box Akt peptide (Table 1). Like the PL peptide, these results imply positive cooperativity and would not be expected to optimally fit the expression for a single binding site complete model (Fig. S4).

**Table 2:** Representative Reported and Predicted PCNA Binding Partners that Contain a PIP Box Motif.

Protein Name	PIP Box Sequence Motif
***Reported Interaction with PCNA***	
DNA-PK	**Q**YF**M**EQ**FY**
Ku70	**YF**VA**L**VP**Q**
MPG	**YF**CMN**I**SS**Q**
***Predicted Interaction with PCNA***	
NEK11	**Q**LL**L**GVD**Y**
PLCγ	**Q**EH**L**ADHE
JAK3	**Q**NP**L**GPD**Y**
SBK1	**Q**LG**L**ALD**F**
PI3K (p85α)	**Q**YL**L**KH**FF**
Akt	**FF**AG**I**VW**Q**

a amino acid residues shown in bold represent PIP Box conserved sequence motif residues.

### Identification of Key Residues Responsible for Molecular Recognition

**Figure 2:**
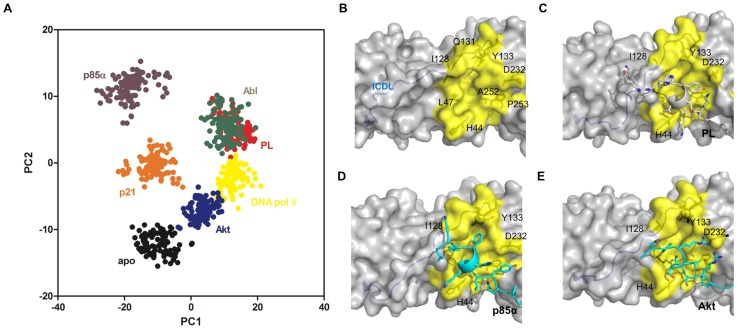
Topology of PIP Box Interaction Site is Ligand-Dependent. (A) Principal component analysis showing clustering of different PCNA conformations (residues 1–257) when in complex with a PIP Box containing peptide ligand at RMSD convergence (4.5–5.0 ns). Rendering of the average topology of the PIP Box interaction site for (B) unligated (apo), (C) PL-PCNA interaction, (D) PI3K (p85α)-PCNA interaction, (E) Akt-PCNA interaction. Residues that outline the interaction site are shown in yellow.

Evaluation of the impact ligand binding has on PCNA was investigated using molecular dynamic simulation analyses. A 5.0 ns simulation was performed on each PCNA-peptide complex and convergence of the PCNA monomer was achieved after 2.0 ns (Fig. S5). Each of the resultant structural assembles represented significant differences in overall structure from the PL-PCNA X-ray crystal structure (Fig. 2). Results further suggested ligand binding to specific PCNA conformations and ligand interactions were optimized through PCNA dynamics to facilitate more favorable complex formation. Regions of PCNA that may help drive a specific conformation and facilitate ligand binding were between residues 81–85, 106–111, and 119–128 (Fig. S6).

Principal component analysis of the trajectory snapshots at convergence (4.5–5.0 ns) between PCNA-peptide complexes identified distinct differences in the overall topology of the interaction site (Fig. 2A). Changes in solvent accessible surface area (ΔSASA) between the unligated and ligated forms of PCNA were calculated using ANCHOR, a tool that analyzes protein-protein interfaces to identify those features that may be useful for small molecule design [31]. Five amino acid stretches were identified to show significant ΔSASA upon ligand binding (Fig. S7). Residues comprising the IDCL were shown to adopt different conformations resulting in slight differences amongst ΔSASA. Residues Leu126 and Pro129 consistently showed the greatest ΔSASA between the unligated and ligated forms of PCNA. These results suggest that these residues may be influential in the overall conformational stability of the ligand, but it is not clear how the residues contribute to the overall binding.

**Figure 3:**
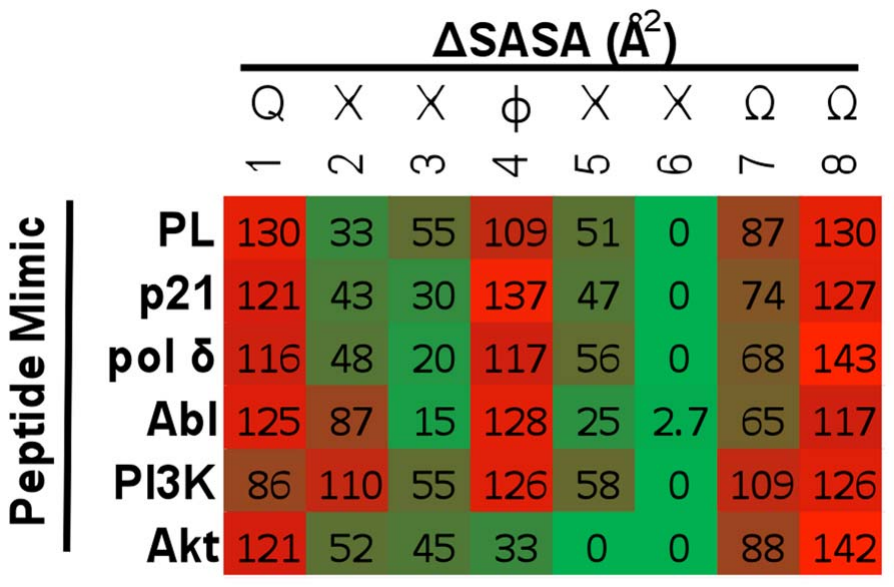
ANCHOR Results of Short PIP Box Peptides Binding to PCNA. Changes in the SASA of the short PIP Box peptide mimic upon ligand binding, as determined by the average trajectory model exported from the molecular dynamic simulations, were calculated using ANCHOR. Values within the heat map indicate ΔSASA between the bound and unbound forms.

Identification of key contact residues at protein interfaces is important to understand molecular recognition features critical for binding (Fig. 3). Ligand residues that contribute to the overall conformational stability of the PCNA-peptide complex can be classified as either anchor or tethering residues [41]. Anchor residues have been characterized as those residues that show a ΔSASA between the unbound and bound ligand greater than 70 Å^2^. For example, the last aromatic residue within the PIP Box of PL was classified as an anchor residue (ΔSASA = 130 Å^2^) that helps in the molecular recognition and conformational stability of the PCNA-PL peptide complex. Several residues that were not classified as anchor residues showed significant contributions to the overall optimized conformation of the PCNA-peptide complex. These residues are classified as tethering residues. For example, in the PL peptide, a lysine residue at position 2 was calculated to have a relatively small ΔSASA (32.7 Å^2^) yet it was predicted to form a hydrogen bond with the backbone of Asp232. The ΔSASA at position 4 (hydrophobic residue of PIP Box) seems to predict PCNA association and suggests that this position may be necessary for the anchoring of the 3_10_ helix commonly observed at this interface. In the absence of a hydrophobic residue at this position, peptide binding to PCNA was not observed [7]. A previous study of PCNA-PL interactions showed that PL residues classified as anchor residues contributed to approximately 65% of the total buried surface of the peptide [13].

**Figure 4:**
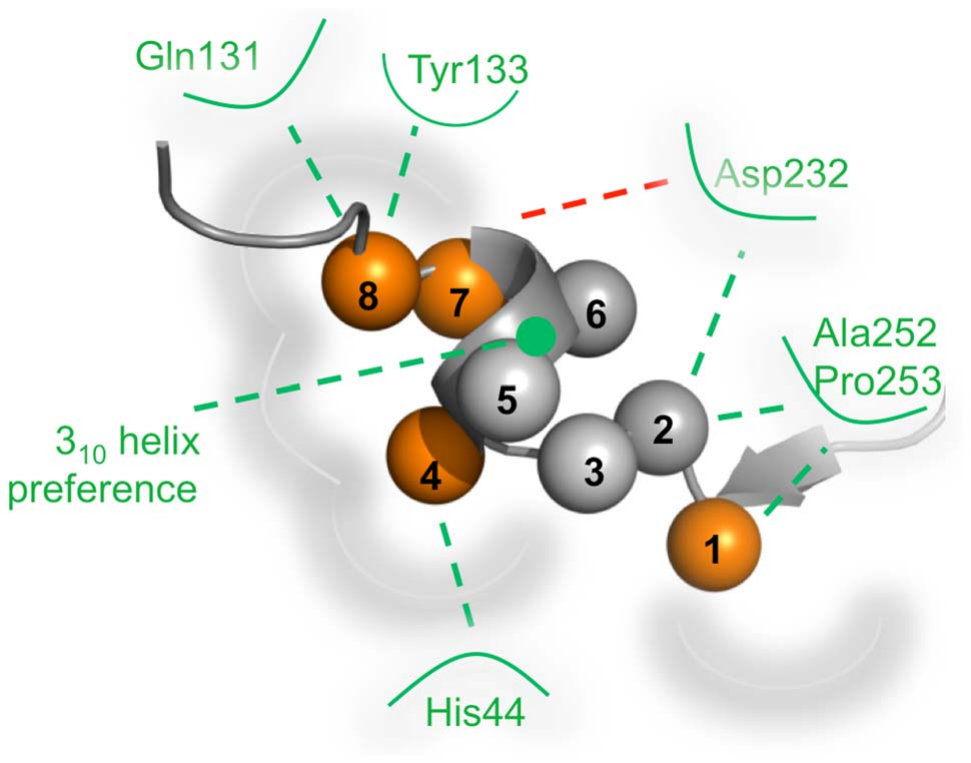
PIP Box Interaction Site Landscape. Spheres represent approximate location of individual residues when bound to PCNA. Residues identified as anchor residues were identified by orange. Key electrostatic and hydrogen bonding interactions shown on perimeter of interaction site and point to those residues considered as tethering.

The formation of hydrogen bonds and electrostatic interactions on the perimeter of the binding site is also critical for the conformational stability of the ligand at the surface binding site and thus a potential important feature to further explore (Fig. 4, S8). Residues that form hydrogen bond or electrostatic interactions are classified as tethering residues. Differences in tethering residues observed between different PIP Box peptides and PCNA could also account for the differences in the overall topology of the interaction site (Fig. S8). For instance, the PL and p21 peptides were shown to form unique interaction site topologies that can be reflected in hydrogen bond differences within the interaction site. The hydroxyl on the tyrosine residue at position 8 of the p21 PIP Box was predicted to form a hydrogen bond with the carboxamide side chain of Gln131 whereas the PL peptide did not demonstrate this interaction. Similar interaction differences were also observed in co-crystal structures of longer PIP Box peptides binding to PCNA previously published [12], [13].

Generated Akt- and PI3K (p85α)-bound PCNA models showed the p85α peptide mimics the other forward PIP Box peptides whereas the Akt peptide demonstrated a different converged binding mode (Fig. 2A). This difference is reflected in the absence of an anchor residue at position 4 despite the fact that there is a hydrophobic residue present (Fig. 3, 4). Comparison of ΔSASA and hydrogen bond interactions between forward and reverse PIP Box containing peptides further supports the notion that the interactions shared between peptides are distinctive. In the case of the Akt reverse PIP Box peptide, a 3_10_ helix was not observed, and the hydrogen bonding interactions between the backbone of Ala252 and Pro253 and the Akt peptide were absent (Fig. S8). Both of these features have previously been described with forward PIP Box containing peptides. Furthermore, the topology of the PIP Box interaction site is distinct from the other complex topologies studied (Fig. 2D, 2E).

### Small Molecule Disruption of PCNA-PL Interactions

Recently, 3,3′,5-triiodothyronine, commonly referred to as T3 hormone, was discovered to bind to PCNA at the PIP Box interaction site through a high-throughput screen [32]. Despite the observations enabled by a co-crystal structure of the T3-PCNA complex, chemical optimization of the hit compound scaffold indicated limitations to developing higher affinity ligands as hypothesized [42]. However, the study did establish the essential features of a 3,5-diiodophenyl ring for binding of the congeners to a defined region of the PIP Box interaction site. This unexpected diversion from the types of side chain interactions observed to date motivated analysis of the binding site and dynamics.

**Figure 5:**
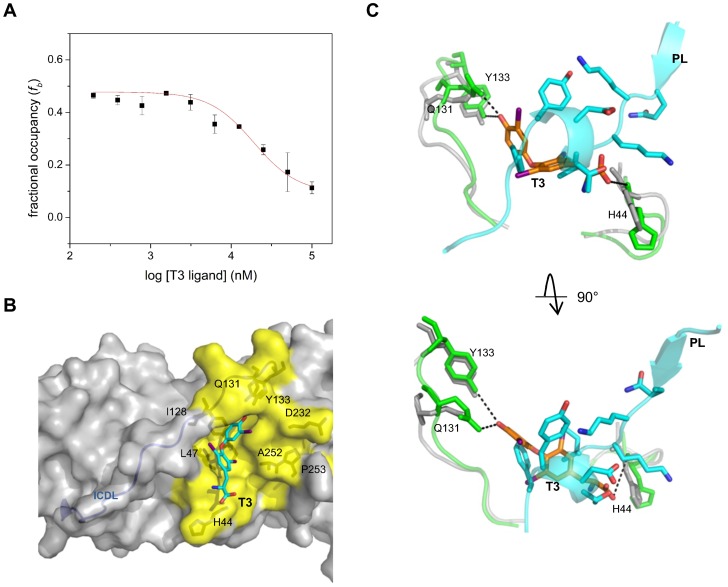
Analysis of T3 Competition with PL for Binding to PCNA. (A) Competition of 50 nM FITC-PL and 1 µM recombinant PCNA protein (monomer concentration) with increasing amounts of T3 ligand. Fractional occupancy, *f_b_*, values (*N* = 4) were derived from anisotropy values using Eq. (3) and represented as mean ± standard error of mean (SEM). Error bars associated with specific data points may be within the data points themselves. (B) Rendering of the PIP Box interaction site for T3 binding to PCNA (PDB 3VKX). Residues that outline the interaction site are shown in yellow. (C) Overlay of PL (cyan) and T3 (orange) ligands to identify features that explain the competitive nature of T3 for binding to PCNA. The top and bottom pose presents a side and top view of the PIP Box interaction site, respectively. Rendering of loop regions Met40-Ser47 and Leu126-Tyr133 are shown for both PL (gray) and T3 (green) conformations. Dashed lines indicate the presence of a potential hydrogen bond.

The capacity of T3 to compete with PL for binding PCNA was hypothesized from structural models (Fig. 5B, 5C). Using the fluorescence polarization assay, the parent T3 ligand exhibited a *K_i_* of 7.8 µM (Fig. 5A, Table 1). Like the p21 12-mer peptide, T3 does not fully displace the PL peptide raising the possibility that both can occupy the same PCNA trimer. These data were fit to the model for incomplete binding to estimate *K_d_* values for each binding event (Table S1, Fig. S4). The observation is also consistent with the primary data presented in the original fluorescence polarization displacement data [32].

A careful inspection of the available data revealed that the topology of the PCNA-PIP Box interaction site showed significant distinctions when T3 is bound compared to the other topologies observed (Fig. 5B). The flexibility of the interface to accommodate iodine atoms deep in the pocket shifted the positioning of residues Met40-Ser47 and Leu126-Tyr133 on the perimeter of the PIP Box interaction site (Fig. 5C). The important hydrogen bonding interaction between the backbone of the hydrophobic residue of the PIP Box and His44 of PCNA previously identified is also observed in the T3 bound crystal structure. This observation suggests that the incorporation of the iodine atoms to T3 may contribute to the observed ∼2 Å shift in the backbone of the His44, allowing a hydrogen bond to form with the carboxylate of T3. Furthermore, residues within the IDCL (Leu126-Tyr133) were shown to adopt a different conformation accommodating the 5-iodine and allowing for a potential hydrogen bond between the side chains of Gln131 and Tyr133 and the 5′-hydroxyl of T3. The results also suggest that displacement of the aromatic residues of PL that interact with the IDCL is sufficient for T3 binding.

## Discussion

Protein-protein interfaces have not been shown to elicit gross topological modulations due to unfavorable energetics associated with conformation change [14], [43]. Instead, small low-energy barrier conformational changes are observed to promote hydrogen bonding and facilitate complementarity between residues [44]. Therefore, protein-protein interfaces are expected to adopt specific conformations that promote or select binding of a ligand [14], [45]. Long distance allosteric effects due to localized binding at these interfaces have not been fully evaluated. These perspectives have yet to be applied to understanding how the basic PCNA trimeric unit is able to recruit a diverse set of protein partners and commute appropriate functional context to DNA damage and replication complexes [1], [6].

The objectives of the study are to elucidate the details of the molecular and dynamic features dictating specificity within the PCNA-PIP Box interaction site. This focus is of central importance to the potential for targeting the site in PCNA and understanding the impact on protein complex formation and stabilization. However, to date there is no clear explanation for how simple docking of a protein transmits a functional consequence to the overall complex. Previous efforts described the critical interactions of tumor suppressor protein p21 and PCNA due to the intimate role in regulating the cyclin-dependent kinase (CDK) complexes. Overall, a relatively high affinity interaction can be observed with a 20-mer peptide sequence derived from p21. However, X-ray crystal structures of the PCNA-p21 complex indicate that interactions extend beyond the consensus PIP Box docking site which likely contributes to the higher affinity. Interestingly, the hybrid 16-mer PL peptide also exhibits an affinity approaching that of p21 despite the moderate similarities in the PIP Box consensus motif. Both the PL and p21 PIP Box peptides were shown to inhibit *in vitro* SV40 DNA replication and suggest that targeting this site can impact PCNA-protein complex formation and biological function [7]. The PIP Box interaction site only contains 8 amino acids, and while these residues are necessary for binding, residues that flank this interaction site may actually contribute more to the overall affinity.

In assessing the diversity of proteins that contain the PIP Box consensus sequence, a strategy was devised to explore the contributions to PCNA recognition. Although the PIP Box is not necessary for PCNA binding of some proteins [46], [47], the general hypothesis is that it serves as an anchor spot and orients the appropriate protein contacts to interact with other surface binding sites. Despite having a conserved PIP Box sequence motif, the peptide mimics exhibited a wide range of affinities. The binding of five different 12-mer peptide mimics of PCNA binding partners was performed (Table 1). A robust fluorescence polarization assay proved accurate enough to report on a range of experimental binding interactions and stoichiometry's within the PIP Box site. Limitations with the assay platform were anticipated as the ligand concentrations approached >100 µM of the competitive ligand due to aggregation of some peptide ligands [48]. Often, extremely hydrophobic patches on the ligands can initiate the aggregation. In the case of competition with Mcl-1, aggregation of the tracer was not observed at higher concentrations whereas aggregation could be readily observed with the p21 12-amino acid peptide beyond 50 µM (data not shown). Longer PIP Box peptide mimics of p21 were also shown to aggregate at similar concentrations [7].

The use of molecular dynamic simulations to extract molecular recognition features allowed for an unbiased approach to optimize or select specific conformations of the PCNA-peptide complexes detected in the fluorescence polarization assay. Molecular dynamics simulations have been used in numerous cases to understand the flexibility of the receptor upon binding of a ligand. Those features that define receptor adaptivity have been used to pursue structure-based drug design efforts [49]–[51]. Using the PL peptide-PCNA X-ray crystal structure as a starting point enabled a comparative basis for model extraction. Structural models reflect the dynamics of PCNA-peptide complexes and are distinct from the binding site shape observed in the X-ray crystal structure. These results are consistent with the expectation that the peptide and small molecule ligands engage distinct topologies from the unligated form of PCNA. More importantly, the molecular dynamics studies illustrated that specific regions of PCNA may become rigidified upon ligand binding. The flexibility of the IDCL and other regions flanking the PIP Box interaction site were also shown to be altered upon ligand binding in a recent NMR analysis of PCNA-peptide interactions [22]. Together, these data are providing significant insights into the dynamics of direct PCNA-ligand interactions.

An important factor in studying the dynamics of PCNA binding is stoichiometry and the potential for cooperative effects or multiple binding equilibriums. PCNA most often exists as oligomers and the trimer appears central to formation of multi-protein complexes *in vivo*. However, factors that govern asymmetry in the association of DNA and protein partners with PCNA trimers are not well understood. Furthermore, the diversity of protein complexes argues that mechanism(s) may exist to regulate association of different proteins to PCNA trimers and stabilize these interactions. This has been pointed out in the case of the FEN1 and p21 competition for PCNA [52]. The variation in Hill coefficients and the data fits prompted additional evaluation of published binding data including binding stoichiometry [38]. Previously, a 20-mer peptide mimic of p21 was shown by ITC and gel filtration chromatography to bind trimeric PCNA with a 1∶1 stoichiometry [7], [53]. More recently, a 12-mer peptide based upon p21 was evaluated for binding with PCNA by ITC and also showed unit stoichiometry [22]. In contrast, the incomplete competitive binding model fit for p21 observed here indicates that different PIP Box ligands influence PCNA conformation and the overall ligand occupancy. Noteworthy is the fact that the PL peptide displays some unique interactions with PCNA reflected here in the Hill slope and recognized in prior reports [7], [13]. Despite the complete displacement indicated by the anisotropy data, the PL peptide data did not fit the generalized model presented in Eq. (6) for a single binding site [26]. ITC data also indicated that PL showed only 2 peptides binding to the PCNA trimer indicating some degree of cooperativity and now likely reflected in the incomplete displacement by the p21 peptide and T3 small molecule (Fig. 1, S4) [7]. Structural variants to accommodate these differences in ligand-PCNA interactions could arise from conformational selectivity of the PIP Box interaction site or IDCL that may regulate formation of distinct PCNA-protein complexes.

The utility in developing small molecule inhibitors of PCNA-protein interactions has been recently highlighted by the discovery of T3 and synthetic congeners, are competitive ligands for the PCNA PIP Box site [32], [42]. These agents have antagonist effects on cellular translesion DNA synthesis in response to drug-induced damage. The crystal structure of the PCNA-T3 complex illustrates that the ligand leverages a protein conformation in order to drive the competition with PL for binding to PCNA (Fig. 5B). Additionally, complete dissociation of the PCNA-PL complex was not observed with any of the competitive ligands. The overall evidence indicates PCNA binding in solution at a single site can impact function, and potential for long distance perturbations in PCNA binding sites can result in asymmetry of PCNA-protein trimer complexes.

Amino acid sequence analysis of the proteome identified 278 human proteins containing a PIP Box conserved sequence motif; however, out of the current list of 144 reported PCNA binding partners, only a small fraction contained a classical PIP Box motif (http://tare.medisin.ntnu.no/pcna/index.php). Table 2 shows a representative list of reported and predicted PIP Box containing peptides. From this list, two new peptide-based PIP Box containing mimics, p85α and Akt were identified to interact with PCNA. Roles for these proteins in the regulation of PCNA assembly onto chromatin have been suggested by previous studies [39], [54]. More interesting is the uniqueness of the Akt PIP Box. This PIP Box is reversed in the primary sequence of the protein. The reverse PIP Box on Akt was shown to compete with PL for binding to PCNA despite dissimilarity in other peptide binding modes from computational models (Fig. 2). The results indicate that the directionality of the PIP Box does impact binding to an extent; however, similar anchor residues are consistent with forward PIP Box containing models (Fig. 2, 3). Amino acid sequence analysis identified that Ku70 also has a predicted reverse PIP Box sequence; however, the importance and relevance of the reverse PIP Box is unknown (Table 2).

### PIP Box Interaction Site is Unlike Most Protein-Protein Interfaces

Much like other protein-protein interfaces, the interaction site is a shallow hydrophobic pocket whose binding partners use electrostatic and hydrogen bonding interactions along the pocket rim to anchor the ligand into the pocket or groove [43], [55]. However, the PIP Box interaction site on PCNA is distinct from other validated protein-protein interfaces. First, the interaction site is adjacent to the IDCL, a highly flexible region of the protein, and the dynamics of this region upon ligand binding have not been explicitly studied. Computational results from molecular dynamic simulations of PCNA-peptide complexes suggest that binding of a ligand stabilizes the backbone conformation of residues 119–128 within the IDCL and the disordered-to-ordered transition of loop may contribute to the overall selectivity of PCNA-protein complexes (Fig. 2, S5). Alanine mutations within the IDCL substantially enhanced overall affinity for numerous proteins involved in DNA repair [56]. The increase in the affinities resulted in increased sensitivity of the mutant yeast organism to DNA damaging agents [56]. Alternatively, a double alanine mutation within the IDCL of yeast PCNA resulted in reduced affinity for FEN1, p15, and DNA polymerases δ and η [57]. Interpretation of these mutational effects is that IDCL residues play a crucial role in the conformation of the PIP Box interaction site.

Conformational effects induced by post-translational modifications and PCNA localization are also hypothesized to alter the PIP Box interaction site conformation. For example, SUMOylated PCNA complexed with DNA specifically enables binding of the Srs2 helicase to carry out its function in DNA replication and repair [58]. More recently, T2AA, an analog of T3, showed selectivity for monoubiquitinated PCNA compared to unmodified PCNA [59]. Therefore, the conformational landscape of the PIP Box interaction site on PCNA may play an important role in ligand selection and complex stability.

Last, 3_10_ helices are not commonly observed at protein-protein interfaces. Recently, the secondary structure elements that appear in protein-protein interfaces of *S. cerevisiae* were characterized. In both hetero- and homo-complexes, a 3_10_ helix was observed less than 5% of the time [60]. The presence of the 3_10_ helix at these PCNA-protein interfaces suggests that this structural element helps to orient the anchor residues to drive desolvation of the interface. A study of p21 and PL peptide binding to PCNA suggests that an aspartic acid residue adjacent to the aromatic residues within the PIP Box motif helps to stabilize the helix [13]. An aspartic acid residue at this position has also been shown to be present only in high affinity peptide-based mimics to PCNA. Further, computational results suggest that the Abl peptide mimic does not form a 3_10_ helix when bound to PCNA and the loss of this feature may contribute to its relatively low affinity interaction with PCNA.

Identification of molecular recognition features can greatly advance understanding of how protein complexes are formed and the extent to which these complexes can be targeted with small molecule modulators. At the heart of many protein complexes are scaffold proteins whose sole responsibility is to recruit, orient, and stabilize complexes. Targeting these scaffolds has remained a challenge since the molecular descriptors influencing ligand binding and selectivity are not well defined. In this study, we used PCNA as a representative scaffold to address the adaptivity of a protein interface to explore ligand-dependent conformational changes. Identification of the features that dictate these conformational changes will aid in the design of potentially selective and/or higher affinity modulators of PCNA-protein interactions.

## Supporting Information

Figure S1 **Binding of N-terminal FITC-labeled PL Peptide to PCNA by Fluorescence Polarization.** Binding isotherm of 50 nM FITC-PL peptide with increasing monomeric concentrations of recombinant PCNA protein. Anisotropy values (*N* = 4) were converted to fractional occupancy, *f_b_*, by Eq. (3) and represented as mean ± standard error of mean (SEM). Data were fit to Eq. (1) to extract a dissociation constant for FITC-labeled PL peptide binding to PCNA. Error bars associated with specific data points may be within the data points themselves.
(TIF)

Figure S2 **Reproducibility of Controls in Fluorescence Polarization Assay.** Anisotropy values (*N* = 24) of 50 nM FITC-PL peptide in the presence and absence of 1 µM recombinant PCNA protein (monomeric concentration) were used to evaluate the quality of the assay platform. Anisotropy values were represented as mean ± standard deviation and a Z′ factor was calculated using Eq. (2).
(TIF)

Figure S3 **Fluorescence Polarization Competition Assay Data and Model Fitting.** Competition of PCNA-PL interactions with short PIP Box peptides using fluorescence polarization. Fractional occupancy, *f_b_*, values (*N* = 4) were calculated from anisotropy values using Eq. (3) and represented as mean ± standard error of mean (SEM). Data were fit to Eq. (4) for determination of *IC_50_ values*. Error bars associated with specific data points may be within the data points themselves.
(TIF)

Figure S4 **Fluorescence Polarization Competition Assay Data using Complete and Incomplete Binding Models.** Competition of PCNA-PL interactions with proposed PCNA ligands using fluorescence polarization. Anisotropy values (*N* = 4) were converted to fractional occupancy, *f_b_*, values using Eq. (3) and represented as mean ± standard error of mean (SEM). If *f_b_* = 0 at the highest concentration of ligand tested, then the data were fit to a complete competition model using Eq. (6). If *f_b_*≠0 at the highest concentration of ligand tested, then the data were fit to an incomplete competition model using Eq. (7). Error bars associated with specific data points may be within the data points themselves.
(TIF)

Figure S5 **RMSD of Cα Atoms over Simulation Time.** The change in RMSD of the alpha carbon atoms from t = 0.0 ps was calculated every 5.0 ps to demonstrate convergence of the molecular dynamic simulations.
(TIF)

Figure S6 **Cα Atoms Fluctuation (RMSF) as a Function of Residue Number.** The change in RMSD of the alpha carbon atoms across all residues calculated every 5.0 ps to demonstrate regions of flexibility.
(TIF)

Figure S7 **ANCHOR Results of PCNA Protein upon Ligand Binding.** Changes in the SASA of the PCNA monomer upon ligand binding, as determined by the average trajectory model exported from the molecular dynamic simulations, were calculated using ANCHOR. Values within the heat map indicate ΔSASA between apo and ligand bound forms.
(TIF)

Figure S8 **Hydrogen Bond Interactions Observed in PCNA-Peptide Molecular Dynamic Simulations.** The absence or presence of a hydrogen bond interaction between the PIP Box containing peptide and PCNA is denoted as a – or a +, respectively. Also shown is whether a 3_10_ helix was also observed in the average trajectory snapshot shown in Fig. 2.
(TIF)

Table S1 **Dissociation Constants for Complete and Incomplete Competition of PCNA Interacting Ligands.**
(DOCX)
