# Supplementary figures and images for: Flexibility of PCNA-Protein Interface Accommodates Differential Binding Partners
Source: PLoS One. 2014 Jul 18;9(7):e102481. doi: 10.1371/journal.pone.0102481 (PMC4103810; doi:10.1371/journal.pone.0102481)

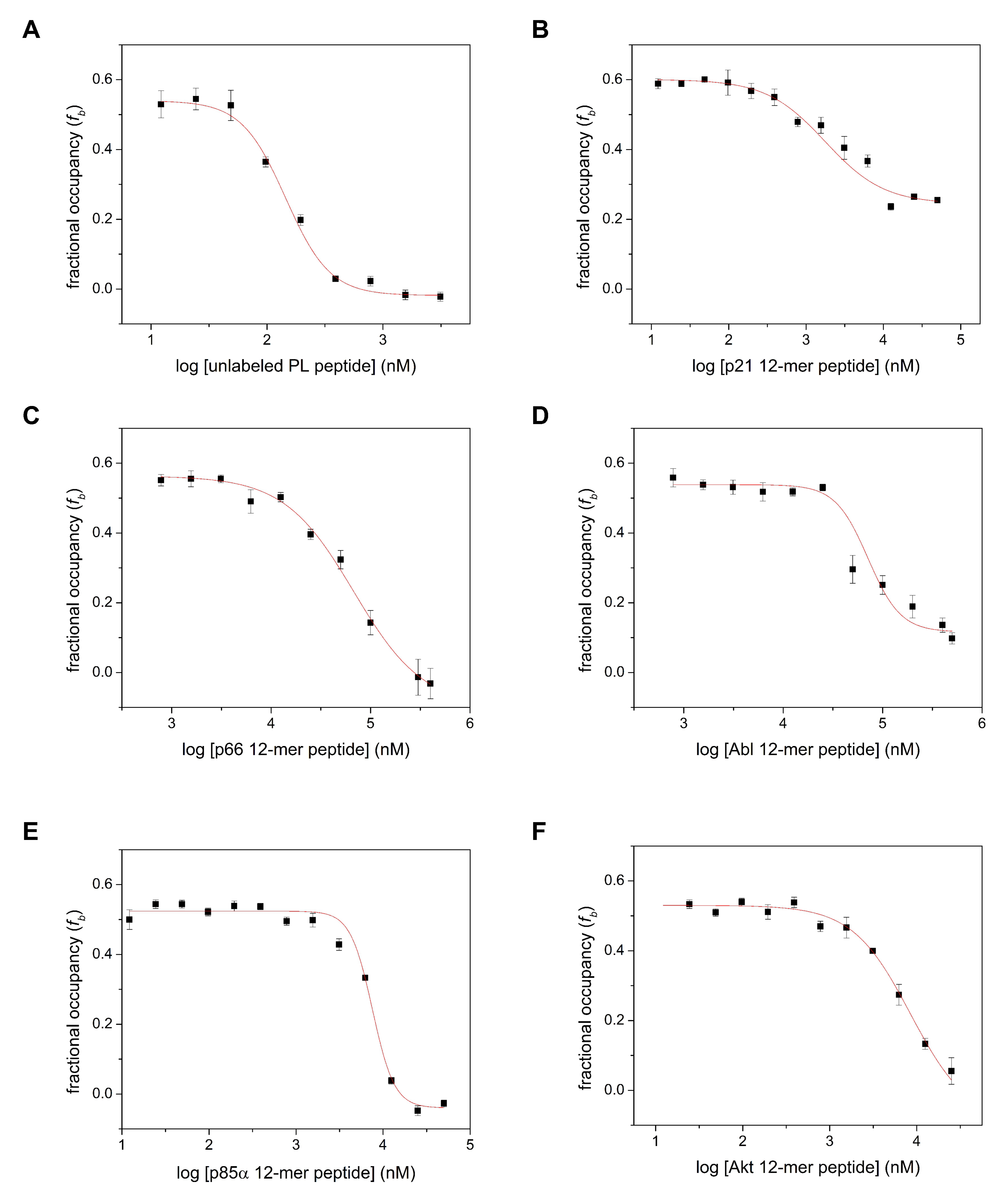

Supplement: Figure S3 — Fluorescence Polarization Competition Assay Data and Model Fitting. Competition of PCNA-PL interactions with short PIP Box peptides using fluorescence polarization. Fractional occupancy, fb, values (N = 4) were calculated from anisotropy values using Eq. (3) and represented as mean ± standard error of mean (SEM). Data were fit to Eq. (4) for determination of IC50 values. Error bars associated with specific data points may be within the data points themselves. (TIF) [file pone.0102481.s003.tif]

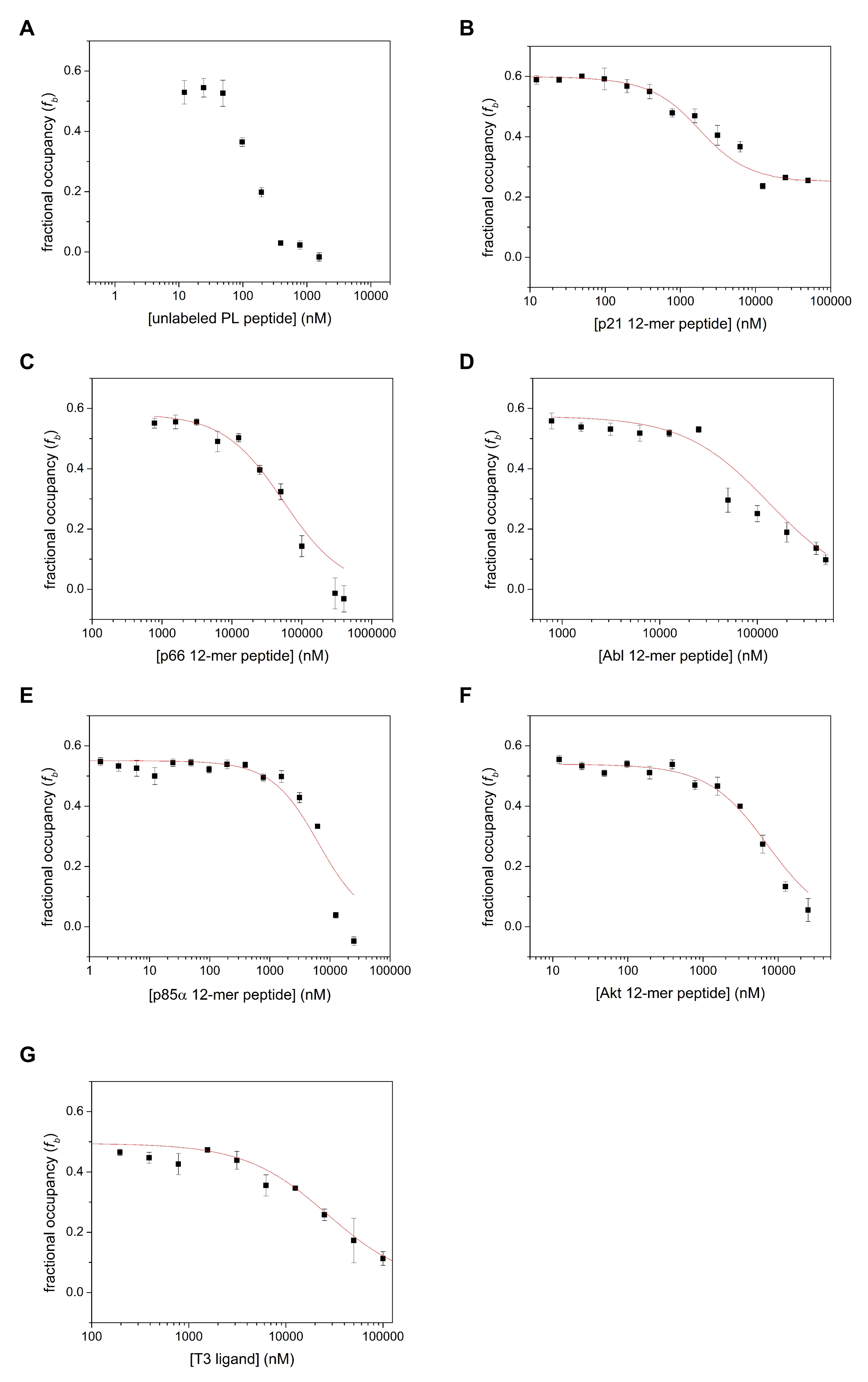

Supplement: Figure S4 — Fluorescence Polarization Competition Assay Data using Complete and Incomplete Binding Models. Competition of PCNA-PL interactions with proposed PCNA ligands using fluorescence polarization. Anisotropy values (N = 4) were converted to fractional occupancy, fb, values using Eq. (3) and represented as mean ± standard error of mean (SEM). If fb = 0 at the highest concentration of ligand tested, then the data were fit to a complete competition model using Eq. (6). If fb≠0 at the highest concentration of ligand tested, then the data were fit to an incomplete competition model using Eq. (7). Error bars associated with specific data points may be within the data points themselves. (TIF) [file pone.0102481.s004.tif]

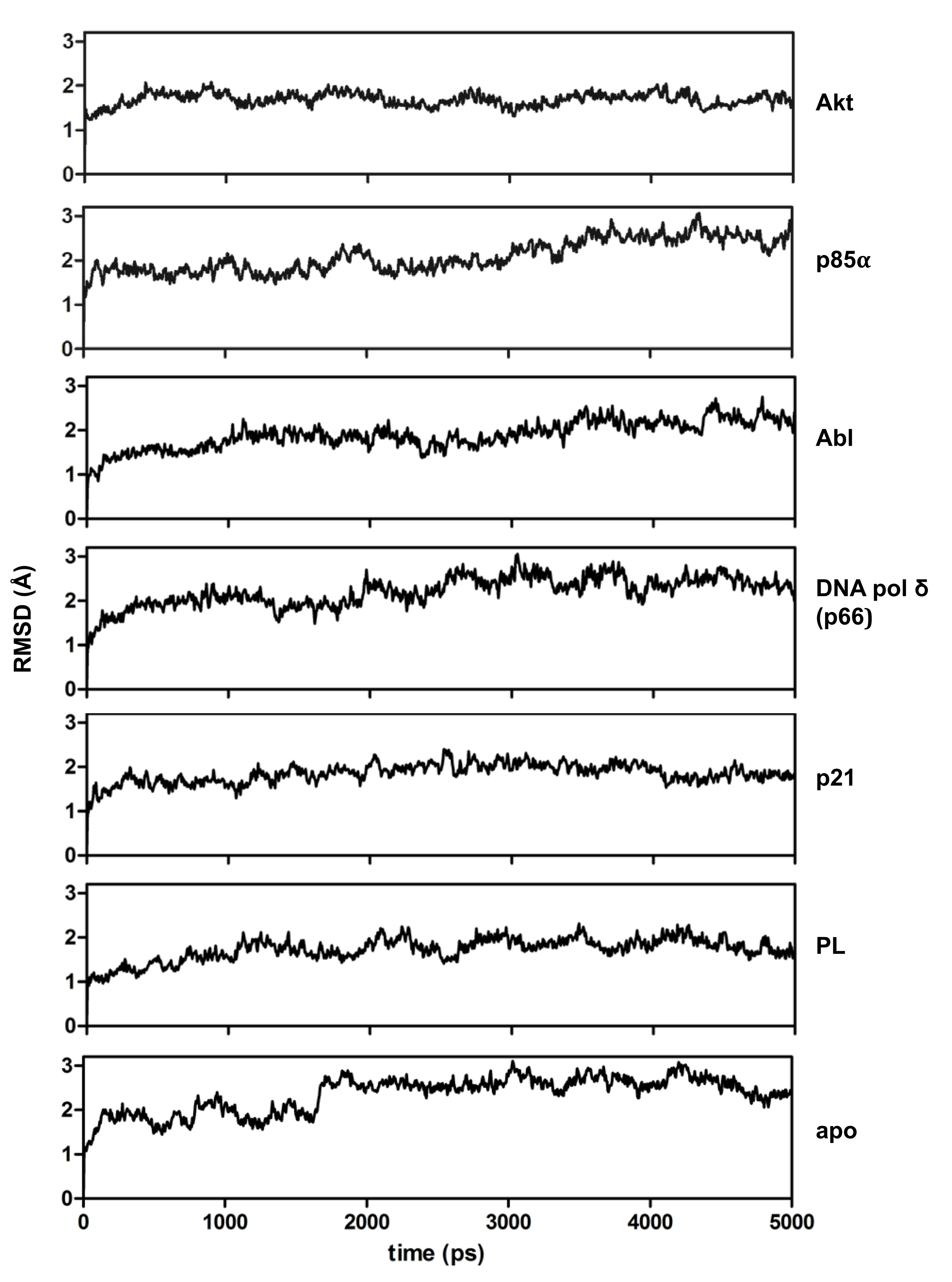

Supplement: Figure S5 — RMSD of Cα Atoms over Simulation Time. The change in RMSD of the alpha carbon atoms from t = 0.0 ps was calculated every 5.0 ps to demonstrate convergence of the molecular dynamic simulations. (TIF) [file pone.0102481.s005.tif]

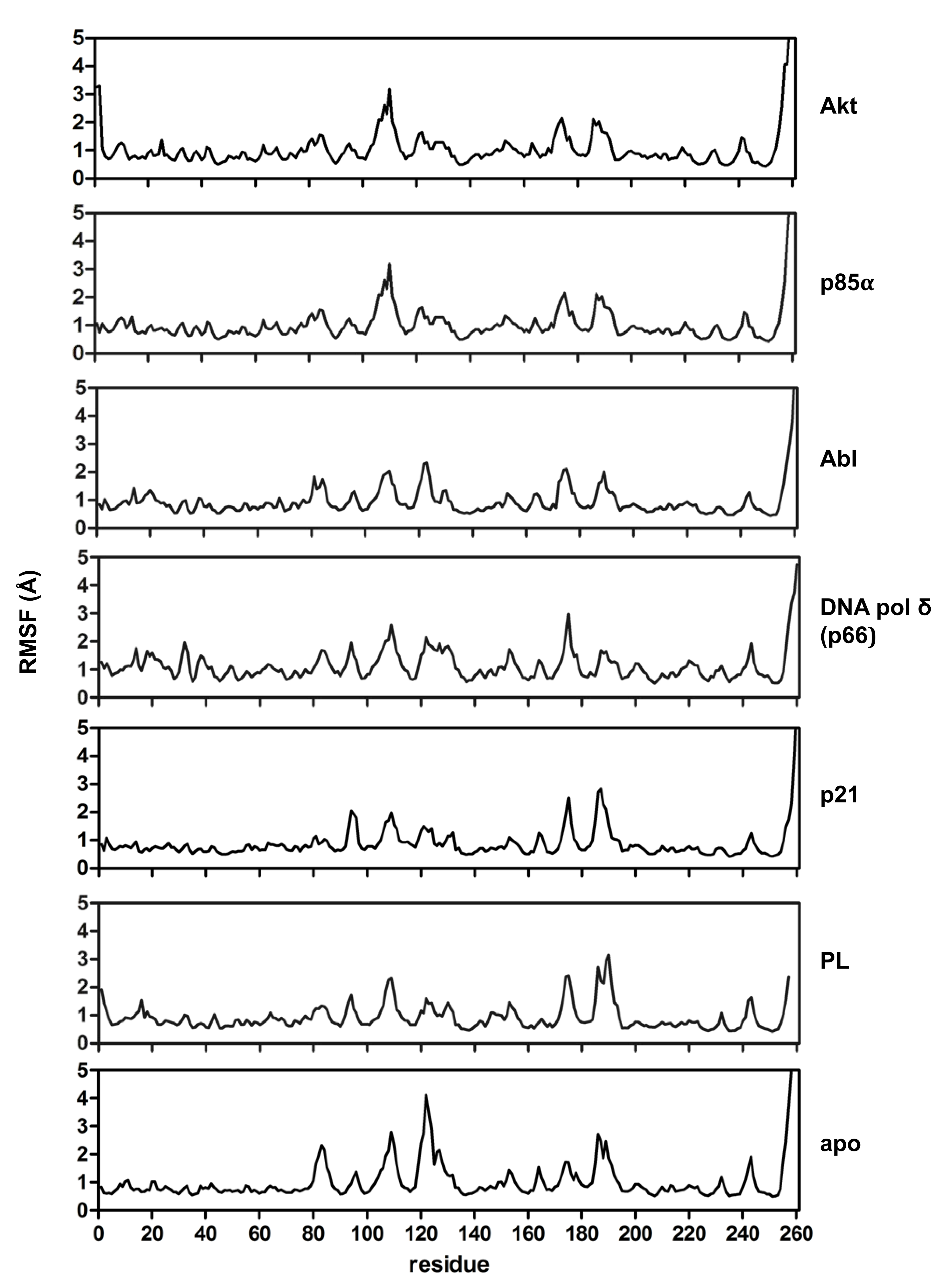

Supplement: Figure S6 — Cα Atoms Fluctuation (RMSF) as a Function of Residue Number. The change in RMSD of the alpha carbon atoms across all residues calculated every 5.0 ps to demonstrate regions of flexibility. (TIF) [file pone.0102481.s006.tif]

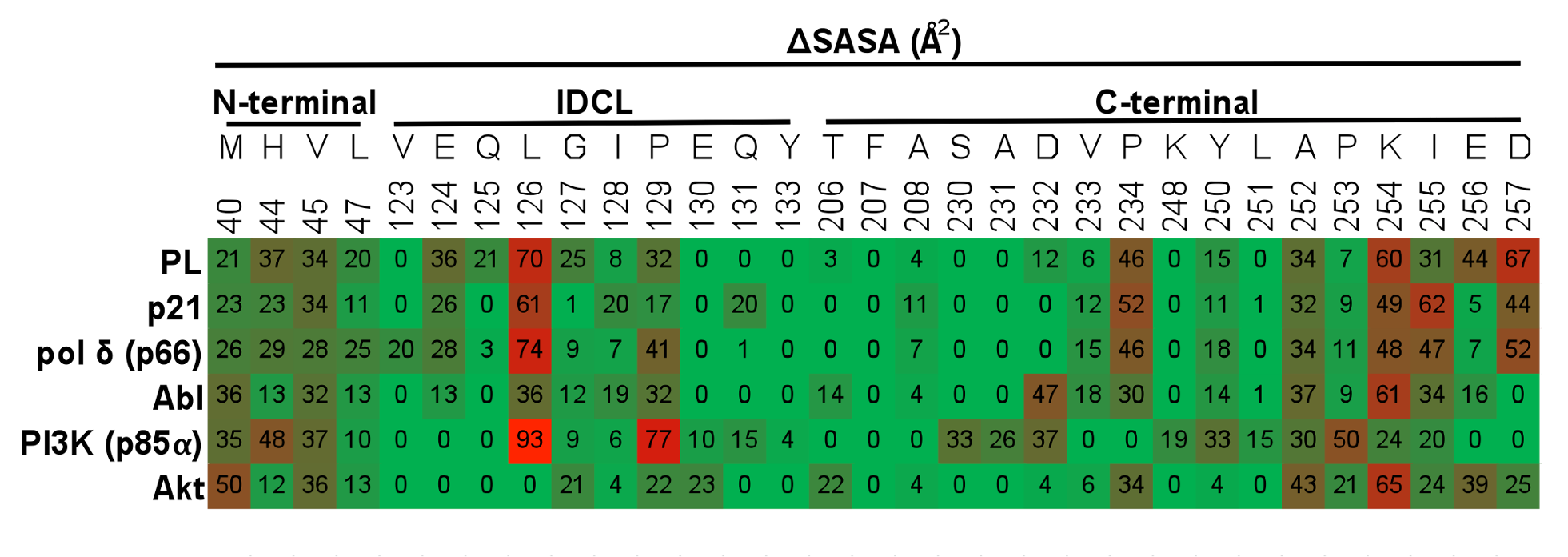

Supplement: Figure S7 — ANCHOR Results of PCNA Protein upon Ligand Binding. Changes in the SASA of the PCNA monomer upon ligand binding, as determined by the average trajectory model exported from the molecular dynamic simulations, were calculated using ANCHOR. Values within the heat map indicate ΔSASA between apo and ligand bound forms. (TIF) [file pone.0102481.s007.tif]

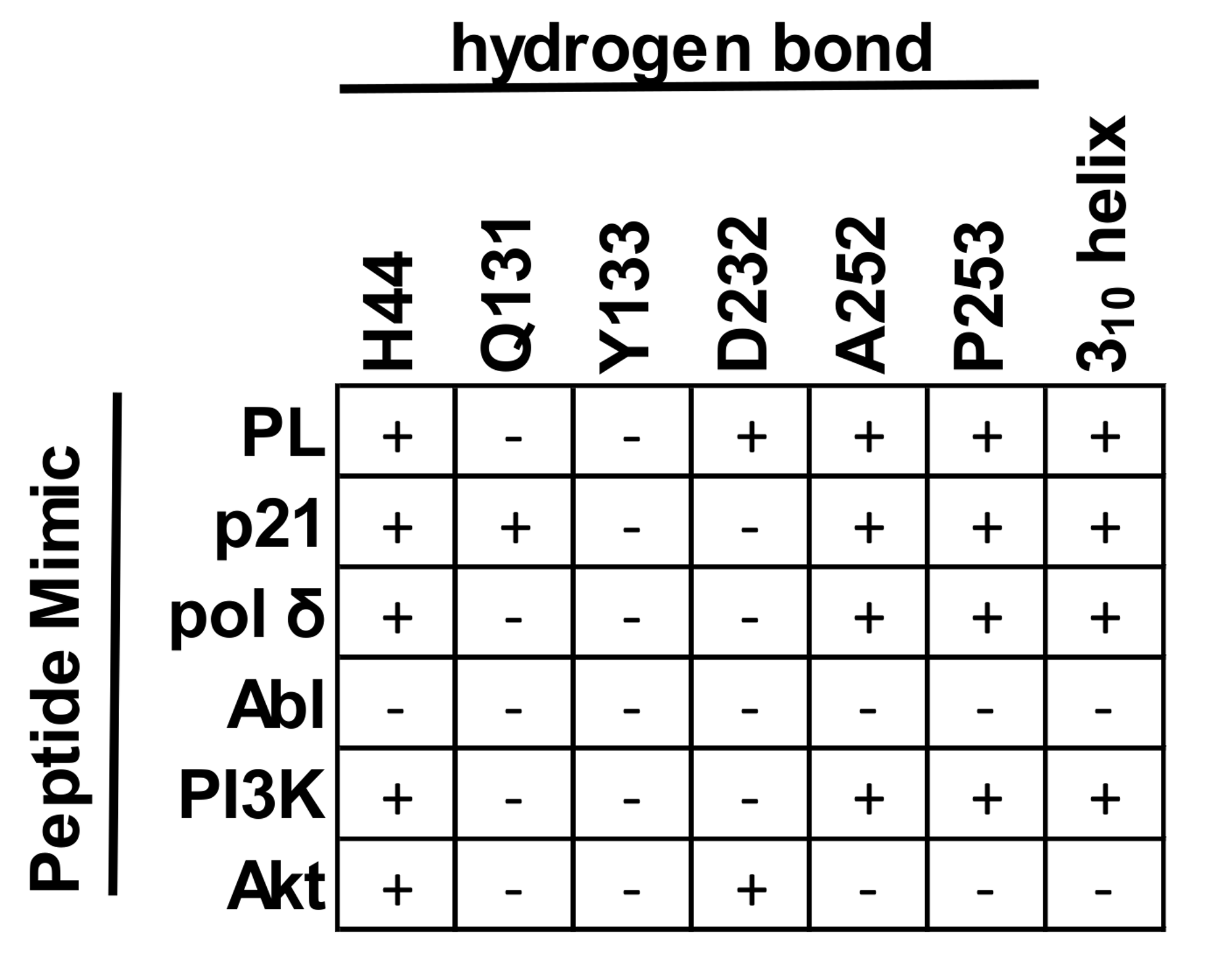

Supplement: Figure S8 — Hydrogen Bond Interactions Observed in PCNA-Peptide Molecular Dynamic Simulations. The absence or presence of a hydrogen bond interaction between the PIP Box containing peptide and PCNA is denoted as a – or a +, respectively. Also shown is whether a 310 helix was also observed in the average trajectory snapshot shown in Fig. 2. (TIF) [file pone.0102481.s008.tif]
