# Supplementary material for: Flexibility of PCNA-Protein Interface Accommodates Differential Binding Partners
Source: PLoS One. 2014 Jul 18;9(7):e102481. doi: 10.1371/journal.pone.0102481 (PMC4103810; doi:10.1371/journal.pone.0102481)
Supplement: Table S1 — Dissociation Constants for Complete and Incomplete Competition of PCNA Interacting Ligands. (DOCX) [file pone.0102481.s009.docx]

**Table S1. Dissociation Constants for Complete and Incomplete Competition of PCNA Interacting Ligands.**

| **Ligand** | **PIP Box Sequence Motif*^a^*** | ***K_d2_* (μM)*^b^*** | ***K_d3_* (μM)*^c^*** |
| --- | --- | --- | --- |
| p21 | RR**Q**TS**M**TD**FY**HS | 0.615 ± 0.251 | 3.1 ± 0.2 |
| DNA polymerase δ (p66 subunit) | NR**Q**VS**I**TG**FF**QR | 20.8 ± 4.4 | **---** |
| Abl | PG**Q**RS**I**SLR**Y**EG | 52.9 ± 14.7 | **---** |
| Mcl-1 | GV**Q**RNHETA**F**QG | *nd* | *nd* |
| PI3K  (p85α subunit) | TL**Q**YL**L**KH**FF**KL | 1.6 ± 0.6 | **---** |
| Akt | HR**FF**AG**I**VW**Q**HV | 2.0 ± 0.4 | **---** |
| T3 | **---** | 13.4 ± 3.2 | ~46.0 |

*^a^* amino acid residues shown in bold represent PIP Box conserved sequence motif residues

*^b^* determined based on Eq. (17) describing complete competition in Roehrl *et al.* [26]

*^c^* determined based on Eq. (27) describing incomplete competition in Roehrl *et al.* [26]

*nd* = no competition detected
